# Overweight and Obesity Among In-School Children and Adolescents (5–19 Years) in Ghana: A Scoping Review of Prevalence and Risk Factors

**DOI:** 10.1155/2024/8895265

**Published:** 2024-10-28

**Authors:** Mustapha Amoadu, Paul Obeng, Jones Abekah Baah, Philomina Acquah, Godfred Cobbinah, Mary Aku Ogum, Jacob Owusu Sarfo, Edward Wilson Ansah

**Affiliations:** ^1^Department of Health, Physical Education and Recreation, University of Cape Coast, Cape Coast, Ghana; ^2^Community Health Nursing Training College, Akim Oda, Ghana; ^3^Nursing and Midwifery Training College, Atibie, Ghana

## Abstract

Overweight and obesity are linked to the severity of infections and the development of chronic conditions among children and adolescents in Ghana. Hence, estimating the current prevalence and its determinants is imperative to guide public health interventions. This review mapped evidence on the prevalence and determinants of overweight and obesity among in-school children and adolescents (aged 5–19 years) in Ghana. Three main databases (PubMed, Central, and JSTOR) were searched for studies conducted in Ghana. Also, the study included only studies published online between 2010 and 2022. The search produced 1214 records, with an additional 23 identified through a search conducted in Google, Google Scholar, the WHO library, HINARI, and institutional repositories. After a thorough screening, 24 records were synthesized. The prevalence of overweight/obesity among the 23,663 in-school children and adolescents in Ghana was 0.5%–47.06%. Females have higher odds of being overweight than males. In addition, lack of nutrition and physical activity (PA) knowledge and low participation in school sports and physical activities exposed in-school children and adolescents in Ghana to overweight and obesity. Consumption of unhealthy foods, late bed, smoking, loneliness, watching television, and playing computer games exposed schoolchildren and adolescents in Ghana to overweight and obesity. There are relatively high levels of overweight and obesity among school-going children and adolescents in Ghana. Addressing sex gaps in PA, ensuring healthy eating, and limiting sedentary lifestyles is the surest way to promote healthy weight among in-school children and adolescents in Ghana.

## 1. Introduction

Overweight and obesity are key and linked to the severity of infections and even the development of chronic conditions. Overweight and obesity are the result of excessive fat accumulation that impairs health or affects normal body functioning [1]. Evidence shows that about 340 million children and adolescents aged 5–19 years were reportedly overweight or obese in 2019 [1]. The condition of being overweight and obese is linked to more deaths worldwide than being underweight. Meanwhile, being overweight and obese, once thought to be issues predominantly affecting high-income nations, are increasingly becoming more prevalent in low- and middle-income countries, especially in metropolitan areas [1, 2].

Due to the rising rates of physical inactivity, and sedentary behaviors linked to obesogenic environments, global populations are now being confronted with overweight and obesity and other contemporary chronic health issues. Consequently, the leading causes of death have shifted from the “conventional” health risks of poverty, such as malnutrition, contaminated water, and poor sanitation, to a rising burden of preventable, noncommunicable diseases including obesity, cancer, and cardiorespiratory conditions [2]. Moreover, the World Health Organization (WHO) lists overweight/obesity as one of the worst health problems of the 21st century and a contributor to many chronic diseases such as heart disease, hypertension, diabetes, and psychosocial issues [3]. Hence, there is an urgent need to protect the younger population using relevant and cost-effective interventions in Africa.

In Ghana, sedentary activities and motorized transportation have supplanted ancient customs including long-distance walking and routine physical labor, especially among urban inhabitants. In addition, like in many sub-Saharan African countries, Ghanaians associate increased body fat with beauty, elegance, prosperity, marriageability, good health, and prestige, whereas thinness is perceived as a sign of impaired health, ugly, and poverty [3]. These factors are likely to increase the prevalence of overweight and obesity exposing the future of the populations and the country's health system to related risk factors for noncommunicable diseases in Ghana.

Children and adolescents are more vulnerable to the health consequences of being overweight or obese due to the possibility of long-term health complications and abject quality of life. Also, an increasing amount of evidence shows that being overweight or obese as a child increases the risk of obesity, physical morbidity, and early death in adulthood due to obesity-induced comorbidity [4]. However, compared to children who remain overweight, those who can achieve a normal weight by adolescence have improved risk factor profiles for cardiovascular and other noncommunicable diseases [1]. Hence, childhood and adolescence are critical periods to acquire fundamental life lessons such as healthy eating and regular physical activity (PA) to maintain a healthy weight for quality of life [5, 6]. Thus, educating children is essential to promoting their well-being. However, there is a need for evidence-based research on contextual factors specific to in-school children and adolescents to effectively establish health promotion programmes for this young population [3, 4]. Although the literature from Ghana is growing, it remains fragmented, necessitating a synthesis of existing information to inform policy and practice. Such evidence synthesis is essential for designing and providing the best interventions to protect and promote the health, well-being, and quality of life of in-school children and adolescents. This approach aims to limit the burden of chronic noncommunicable diseases and associated socioeconomic challenges on the country [4]. Hence, this review maps and synthesizes evidence on the prevalence and risk factors associated with overweight and obesity among in-school children and adolescents (5–19 years) in Ghana to inform policy and practice.

## 2. Materials and Methods

We conducted this scoping review based on Arksey and O'Malley guidelines [7] by (1) identifying and stating the research questions, (2) identifying relevant studies, (3) selecting the studies, (4) collecting the data, (5) data summary and synthesis of results, and (6) consultation. The following questions guided this review: (1) What is the prevalence of overweight and obesity among in-school children and adolescents in Ghana? (2) What are the risk factors for overweight and obesity among in-school children and adolescents in Ghana?

Three main databases (PubMed, Central, and JSTOR) were searched for relevant literature. Medical subject heading (MeSH) terms were utilized for the search in PubMed and refined for the search in other databases. The search strategy is presented in Table 1, with exclusion and inclusion criteria. The keywords in Table 1 were moved to the MeSH, and adapted to fit other databases (Central and JSTOR). The search for relevant data lasted for 4 months (August to November 4, 2022) and was supported by a Chartered Librarian at the Sam Jonah Library, University of Cape Coast, Ghana. The authors scrutinized the records obtained, and the Mendeley software was used to remove duplicates. The WHO Library, HINARI, Google Scholar, Google, and institutional repositories of universities in Ghana were searched for additional records. Furthermore, reference lists of eligible records were checked for other relevant articles. The search included only articles published from 2010 to 2022. The last search for relevant literature was conducted on November 4, 2022. Three authors (MA, PO, and GC) scrutinized the identified records against the inclusion criteria and finally settled on studies that were included in the study. Details extracted during the data charting process include authors and year, study design, population, sample size, prevalence, risk factors, and study recommendations. Three of the authors (M.A., P.O., and J.A.B.) extracted the data independently and later met to compare the results extracted and also settled any discrepancies in the extracted data. When the authors could not agree on discrepancies in the extracted data, an independent researcher (P.A.) was consulted to settle the differences. All authors reviewed the final extracted data before thematic analysis and data synthesis were conducted. The details of the extracted data are presented in Table 2 (see supporting file). Finally, thematic analysis and synthesis were performed, and the results were presented.

## 3. Results

### 3.1. Search Results

**Figure 1:**
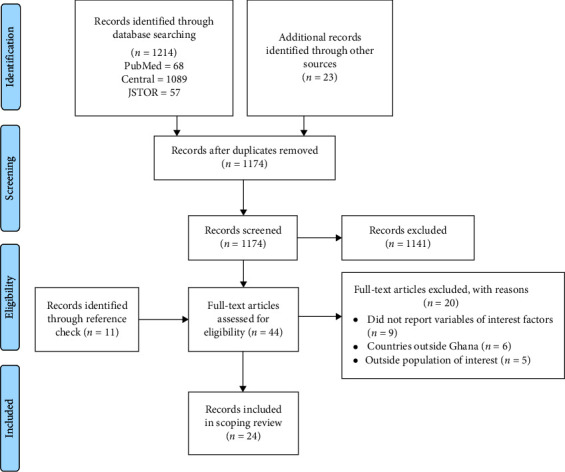
PRISMA flow diagram of records and screening process.

The search conducted in the three main databases produced 1214 records. Also, the search through Google Scholar, Google, and institutional repositories produced 23 records. After removing duplicates (63) using the Mendeley software, 1174 records were available for screening. Furthermore, 1141 abstracts and records not relevant for this scoping review were initially excluded from eligibility checks. In addition, 11 papers were retrieved through reference checking. In all, 44 full-text records were eligible for further screening. Finally, 24 full-text records were included in the thematic analysis and synthesis (see Figure 1 for details on the screening process in the Preferred Reporting Items for Systematic Reviews and Meta-Analyses [PRISMA] flow diagram).

### 3.2. Study Characteristics

The majority (23) of the reviewed studies were cross-sectional surveys with only one being a longitudinal study (Annan, 2021). Reviewed studies sampled 23,667, an average of 986, in-school children and adolescents. Most (16) of the studies focused on basic school children and adolescents, while five focused on high school students and the remaining three studied students from basic to senior high school. The details of study characteristics of included studies are presented in Table 2.

### 3.3. Prevalence of Overweight and Obesity

The prevalence of overweight/obesity in the reviewed studies ranges from 0.5% [8] to 47.06% [9], with female in-school children and adolescents having a higher prevalence [8, 10–13]. For instance, female school children and adolescents recorded a higher prevalence of overweight, ranging from 10.4% to 15.6%, whereas their male counterparts recorded a relatively lower prevalence of 4.5%–7.2% in studies comparing female and male prevalence [8, 12, 13]. In addition, eight studies reported a prevalence of obesity below 10% [8, 14–20]. In contrast, Amoh and Appiah-Brempong [9] (47.06%), Annan-Asare et al. [10] (27%), Mogre et al. [21] (17.4%), Akornor [22] (15.3%), Aboagye et al. [23] (13.7%), and Osei [24] (11.42%) recorded a relatively higher prevalence above 10% of obesity among in-school children and adolescents in Ghana. Three reviewed studies reported a combined prevalence of overweight and obesity between 10.5% and 17% [25–27]. Moreover, Akornor [22] (11.1% overweight and 13.7% obese) and Aboagye et al. [23] (9.59% overweight and 11.4% obese) reported a higher prevalence of obesity than overweight.

### 3.4. Risk Factors of Overweight and Obesity

Reviewed studies have shown that poor dietary knowledge and practices are risk factors for overweight and obesity among in-school children and adolescents in Ghana [14, 23, 28, 29]. For instance, fast food consumption [10]; high intake of processed meat, fried foods, and sugary foods [30]; less consumption of fruits and vegetables [22]; eating and drinking beverages between meals [23]; and taking snacks before bed [15] are risky dietary practices associated with overweight and obesity among school children and adolescents.

Furthermore, included studies have reported that physical inactivity is a risk factor for being overweight. Thus, a lack of or low participation in sports [26] and physical activities [12, 14, 22, 24, 27–29] among school children and adolescents may make them physically inactive, and hence predispose them to overweight and obesity. In addition, children and adolescents not biking or walking to school are linked to being overweight in Ghana [13]. For instance, children and adolescents who use cars or motorbikes to school are more likely to be overweight or obese [17].

Moreover, evidence from the reviewed studies indicates that late bed [21], daily television viewing [9, 18, 21, 22], and playing computer games [9, 17, 18] are risk factors for overweight/obesity. Younger children and adolescents (below 10 years) [14], female school children and adolescents [10, 14, 15, 18, 22, 24, 25], male school children and adolescents [22], children and adolescents of mothers with tertiary education [26] or higher education [17, 22], and school children and adolescents of parents with high socioeconomic status [11, 26] may be more vulnerable to overweight/obesity. Moreover, children and adolescents attending public schools [14] and private schools [17, 18, 20, 26, 31] are all vulnerable to being overweight and obese. Finally, a reviewed study reported that school children and adolescents who smoke and feel lonely are likely to become obese or overweight [8].

## 4. Discussion

### 4.1. Summary of Findings

There is a high level of obesity and overweight among in-school children and adolescents in Ghana. It was revealed that being female, physical inactivity, high socioeconomic status, daily watching of television and playing video games, loneliness, attending private school, and poor dietary practices are making in-school children and adolescents more vulnerable to obesity and overweight in Ghana.

### 4.2. Prevalence of Overweight/Obese

Evidence in this review shows that school children and adolescents in Ghana are significantly more likely to be overweight and obese than predicted (11% overweight/obese by 2025) [2, 13]. If this trend is not controlled then Ghana's aim to achieve “no increase in obesity levels” by 2025 for infants and children may not be realized [2]. We believe Ghana is far from achieving this target by 2025. This is because of the higher rates of the conditions that are most likely to produce compromised health and well-being for children and adolescents but more importantly during adulthood. The relatively high level of overweight and obesity among the in-school population indicates higher levels of obesity-related comorbidities such as impaired glucose tolerance, Type 2 diabetes, hypertension, and hepatic steatosis. Ghana's already compromised healthcare system needs to make provision for a significant increase in obesity-related comorbidities.

The high prevalence of obesity and overweight among in-school children and adolescents in Ghana coupled with poor eating practices indicates that the school environment needs to be reoriented to encourage healthy food choices and lifestyles. Though boys and girls are experiencing higher levels of overweight/obesity over time, body composition measurements revealed girls are proportionately heavier than boys due to adiposity differences and growth spurt. In another vein, higher percentages of overweight/obesity among Ghanaian school girls may be attributed to gender roles that require girls to be physically less active and boys to be more active physically (e.g., boys participating in higher energy expending roles/activities) and cultural desirability wherein being overweight is an admired trait and perceived as a sign of wealth, prestige, and beauty, particularly in girls [18, 20]. Moreover, a lack of enthusiasm and body confidence can be ingrained in children and adolescents through exposure to rigid gender stereotypes around boys versus girls' activities, and a failure to modify sports to accommodate girls in schools can affect attitudes toward PA into adulthood [32]. Nevertheless, despite the impending danger of continued high levels of childhood overweight/obesity in Ghana, effective public health interventions such as health education and screening may help to reduce the hazards and their attendant complications into adulthood, to promote health and quality of life.

### 4.3. Risk Factors of Overweight/Obesity Among In-School Children and Adolescents

This review showed that there is a lack of nutrition knowledge which may compromise healthy food choices and eating among school children and adolescents. Perhaps, schools lack methods or programmes of instruction that are focused on enhancing lifestyle and dietary choices. It is a belief that such programmes and methods are healthful and that their deficiency could lead to an insufficient understanding of nutritional practices among school children and adolescents in Ghana [30, 33]. Moreover, there is a possibility that parents, schools, and the media do not engage school children and adolescents sufficiently in healthy eating, limiting the children and adolescents' nutritional understanding and associated practices. The sale of “fast foods” at school cafeterias exposes children and adolescents in particular to eating unhealthy diets thereby promoting body weight gain [18]. Even the aggressive marking of these foods products and lack of knowledge about the negative health impacts of these high-energy foods make children and adolescents attracted to eating such unhealthy products such as snacks which they may eat as they watch television [22].

Children and adolescents attending both public and private schools are vulnerable to being overweight and obese due to several factors. Perhaps both groups potentially have increased access to unhealthy, calorie-dense foods in Ghana [18, 20]. In addition, PA levels may be low across both school types, either due to inadequate physical education programs or limited recreational spaces [26]. Socioeconomic factors also play a role, as children and adolescents in private schools might have more access to high-calorie foods, while those in public schools may face food insecurity leading to poor nutritional choices when food is available [17, 20, 31]. However, more studies reported a higher prevalence in private schools compared to public schools. This discrepancy may be attributed to several reasons. Children and adolescents in private schools often have greater access to unhealthy, calorie-dense foods and beverages available through school canteens and vending machines, coupled with more disposable income to purchase these items [17, 26]. Private schools may have more extracurricular activities focused on academics and technology, leading to less time for PA. Maybe much attention needs to be paid to physical space and facilities that promote PA in private schools. Also, lifestyle factors such as increased screen time and sedentary behaviors are prevalent among children and adolescents in private schools, who may spend more time on digital devices both for education and recreation [17, 18, 20]. Besides, children and adolescents from higher-income families who attend private schools may have different dietary habits, including higher consumption of fast food and processed snacks, contributing to a higher risk of overweight and obesity [26, 31].

Also, increased sedentary behaviors in school children and adolescents have been associated with an increase in the prevalence of childhood overweight/obesity, primarily as a result of indoor activities including playing video games, watching television, and using the internet which are common in private schools. Due to rapid urbanization, there are fewer open playgrounds in private schools and neighborhoods, which makes it dangerous for children and adolescents to also play outside [20]. Moreover, there is a continual emphasis on academic achievement at the expense of children and adolescents PA [17, 27, 31].

### 4.4. Implications for Policy and Practice

Obesity and overweight are mostly preventable. The choice of healthier foods and frequent PA should be the simplest one (the choice that is most accessible, available, and inexpensive) to avoid or reduce overweight and obesity among children and adolescents in Ghana. This could be made possible by supportive school environments and communities [34]. Making efforts to ensure better access and investment in PA facilities in and out of school as well as a change in sociocultural norms could, therefore, be the starting point for addressing the gender gap in physical inactivity and associated health complications [34]. There is also the need for partnership between relevant stakeholders including the Ghana Ministry of Education, Ghana Ministry of Health, schools, parents, and community leaders in designing and implementing childhood obesity prevention interventions to ensure their cultural relevance and sustainability. In addition, one strategy is to alter the built environment and offer public exercise facilities. Evidence shows that walkable neighborhood helps to increase the PA rate and reduce the gender gap in PA [32].

Education of students is one of the primary ways schools may improve the health of their children and adolescents. To teach school children and adolescents the skills they need to choose and sustain healthy lifestyles, nutrition, and PA classes can be incorporated into the curriculum as core classroom topics, including physical education and after-school programmes [34]. School physical education should emphasize engaging in high-quality and frequent activity, in addition to providing evidence-based nutrition. Schools can stop selling unhealthy meals and add better food options to their cafeteria menus to enhance nutrition and promote healthy eating. Schools should create secure paths for students to walk or bike to school and encourage active recess to increase PA. A supportive school environment that promotes healthy food choices, and PA, while limiting sedentary lifestyles among in-school children and adolescents is one of the surest ways to promote healthy weight among this vulnerable population. This will help to promote healthy living and quality of life in both childhood and adulthood.

### 4.5. Implication for Future Studies

To address the high prevalence of overweight and obesity among school children and adolescents in Ghana, further research is essential. High-quality studies such as randomized controlled trials and longitudinal studies should explore the socioeconomic variables contributing to these conditions. Moreover, longitudinal cohort studies are recommended to understand the causal relationships between identified risk factors and the development of overweight/obesity over time. Notably, higher rates are observed among private school children and adolescents from higher socioeconomic backgrounds. Future research should dissect the specific socioeconomic, cultural, and environmental factors (e.g., built environment and food environments) driving these differences. This detailed understanding will aid in designing effective interventions to curb the rising prevalence of overweight and obesity among school children and adolescents in Ghana.

### 4.6. Limitations in This Review

This review has yielded valuable insights into the nutritional status and contributing factors among school-going children and adolescents. Nonetheless, there exist certain limitations to this investigation. Primarily, our literature review was confined to English-language publications for practical considerations, potentially resulting in oversight of pertinent literature in other languages. Moreover, due to constraints of specific databases, our search was limited to just three databases and manual searching; consequently, there is a possibility that relevant literature was overlooked in our review. Most of the included studies are cross-sectional surveys, which may be susceptible to response bias, as they often rely on self-reported measures. This response bias could potentially affect the findings of this review and limit the generalizability of the results. In addition, the methodological quality of the included studies was not appraised. Therefore, caution should be exercised when drawing conclusions and generalizing the findings of this review. Also, most studies that used children and adolescent populations failed to provide subgroup analysis. Hence, future studies should address this gap in evidence.

## 5. Conclusion

We found in this scoping review relatively high levels of overweight and obesity among school-going children and adolescents in Ghana. Notably, females exhibit a higher susceptibility to overweight or obesity compared to their male counterparts. Our analysis revealed that various factors contribute to this trend, including inadequate knowledge of nutrition and PA, low engagement in school sports and PAs, consumption of unhealthy foods, irregular sleep patterns, smoking, feelings of loneliness, excessive television viewing, and extensive computer gaming. Interestingly, these risk factors are more pronounced among children and adolescents attending private schools and those from higher socioeconomic backgrounds. Addressing these identified risk factors is imperative for mitigating the prevalence of overweight and obesity among children and adolescents in Ghana. By implementing targeted interventions to promote healthy lifestyles and behaviors, Ghana stands to enhance its prospects of achieving Sustainable Development Goal (SDG) 3, which aims to ensure good health and well-being for all by 2030. The strategic mitigation of these factors is imperative for fostering a healthier and more resilient younger generation in Ghana.

## Figures and Tables

**Table 1:** Planned search strategy.

Item	Search strategy
Database	PubMed, Central, and JSTOR
Language filter	English
Date filter	2010–2022
Spatial filter	Ghana
Keywords	1. Overweight OR Obese OR obesity OR obese OR nutritional status OR fat OR fatness OR adiposity OR fatty OR body size. 2. Prevalence OR Percentage OR Proportion 3. Risk factors OR determinants OR causes 4. School Children OR child OR childhood OR infants OR toddlers OR adolescents OR adolescence OR Junior high school students OR senior high school students
Inclusion criteria	The paper should be 1. A peer-reviewed article, thesis, or dissertation 2. A paper published from 2010 to 2022 and was conducted in Ghana only 3. Conducted among school-going children and adolescents 4. Conducted among school children and adolescents aged 5–19 years old; and 5. Conducted on prevalence and risk factors of overweight and obesity.
Exclusion criteria	The paper should be 1. A study written in other languages aside from English language 2. A review, abstract, minute, commentary, letter to editors, and literature reviews

## Data Availability

All data generated or analyzed during this study are included in this published article (and its Supporting Information files).

## Supporting Information

Table 2 presents the extracted data from the included studies.
