# Supplementary material for: Overweight and Obesity Among In-School Children and Adolescents (5–19 Years) in Ghana: A Scoping Review of Prevalence and Risk Factors
Source: J Obes. 2024 Oct 28;2024:8895265. doi: 10.1155/2024/8895265 (PMC11535413; doi:10.1155/2024/8895265)
Supplement: Supporting Information — Additional supporting information can be found online in the Supporting Information section. [file 8895265.f1.docx]

Table 2: Extracted data from included studies

| Author & Year | Purpose of the study | Design/methods | Population | Sample  size | Prevalence of overweight/Obese | Risk factors |
| --- | --- | --- | --- | --- | --- | --- |
| [6] | To assess overweight and obesity and associated factors in school-going adolescents | Cross-sectional survey | School children (13-15 years) | 5613 | Overweight (10.4% girls and 3.2% boys) and Obesity (0.9% girls and 0.5% boys). | Smoking cigarettes and loneliness. |
| [7] | To determine the prevalence and risk factors of obesity among Senior High School  Students in the Adansi  North District of Ghana. | Cross-sectional survey | Senior high school students (12-19 years). | 306 | 47.06% obesity. | Playing computer games, TV watching, residing in urban and living in high- or low-income families. |
| [8] | To examine the prevalence and correlates of obesity. | Cross-sectional survey | Junior high students (11-15 years) | 260 | Obesity (27% female and 26% males) | Female sex and fast-food intake, |
| [9] | To determine the prevalence of obesity among children in the University Primary School, Legon. | Cross-sectional survey | School children (5-15 years). | 270 | 10.9% obesity (15% females and 7.2% males). | High socio-economic background. |
| [10] | Aimed at estimating overweight and determining the pattern and level of physical activity among senior high school students in the Accra Metropolis. | Cross-sectional survey | High school students (15 -19 years). | 444 | 11.7% overweight (15.6% females and 4.5% males). | Low physical activity |
| [11] | To examine the prevalence and risk factors for underweight, overweight and obesity. | Cross-sectional survey | School children (11-17 years) | 6155 | Overweight (13.3% females, 6.7% males). Obese (1.5% females and 0.8% males). | Younger adolescents (<12 years), not walking/biking |
| [12] | To determine the relationship between nutrition and physical | Cross-sectional survey | School children (8-13 years) | 591 |  | Lack of adequate nutrition knowledge and physical activity. |

|  | activity knowledge and using BMI-for-age of school-aged children. |  |  |  |  | Younger children (8-10 years), female male, and those in public schools. |
| --- | --- | --- | --- | --- | --- | --- |
| [13] | To evaluate the prevalence of overweight, obesity and underweight and its related factors among school-aged children and adolescents. | Cross-sectional survey | Basic and senior high school student | 1004 | Overweight (13.8% in basic school students and 12.6% in senior high). Obesity (8.8% in basic school and 8.9% in senior high). | Female sex were more likely to be overweight and obese.  Taking snacks before bed, Watching TV, sleeping during leisure periods, and bicycling to school are protective factors. |
| [14] | To determine the prevalence of obesity and overweight among students in the Kumasi metropolis | Cross-sectional survey | Students (10-20 years) | 500 | 12.2% overweight and  0.8% Obese |  |
| [15] | Determinants of childhood obesity among basic school children aged 6 – 12 years in Tamale Metropolis | Cross-sectional survey | Basic school children (6-12 years) | 400 | 9.8% overweight and obese 7.5% | Children with parents who have higher education levels. Going to school by car/motorbike, playing computer games, carrying food to school and eating from the school canteen, attending private school. |
| [16] | Prevalence and determinants of childhood obesity among school-age children in the Sunyani Municipality | Cross-sectional survey | School children (6 and 18 years) | 423 | 9.7% overweight and obese 6.4% | Attending private school, being a female, playing digital games, spending more time watching TV |
| [17] | To assess the lifestyle habits, macronutrient intakes, and obesity prevalence among adolescents in some | cross-sectional survey | Senior High School  adolescents (13-19 years) | 272 | 15.8% overweight and 8.5% obese | Female sex and adolescents who did not meet the RDA for calories. |

|  | selected Senior High Schools in rural communities in Ho Municipality |  |  |  |  |  |
| --- | --- | --- | --- | --- | --- | --- |
| [18] | To determine the prevalence of obesity and its sociopsychological effects on primary school children in the Cape Coast Metropolis. | Cross-sectional survey | Primary school pupils (9-15 years) | 341 | 5% obesity | Attending private school, being a male adolescent, age. |
| [19] | To assess the prevalence of childhood overweight/obesity and thinness among school-aged children in Tamale | Cross-sectional survey | School children (5-14 years) | 218 | 29.8% overweight and  17.4% obese | Late bed times and Television viewing. |
| [20] | To investigate childhood obesity and its associated factors among primary school pupils within Tema Metropolis. | Cross-sectional survey | Upper primary school pupils | 363 | 15.43% obesity | Consuming fewer fruits and vegetables, consuming more sweetened drinks, or substituting fruits and vegetables with high-fat and refined cereals. Eating breakfast consistently. Watching television each day. Performing fewer physical activities. Coming from a low economic stratum. |
| [21] | To determinethe prevalence of undernutrition and overweight/obesity and its associated factors among children aged 6–12 in the South Tongu District, Ghana. | Cross-sectional survey | School children (6-12 years) | 423 | 11.1% overweight and  13.7% obese | Parents with formal education, consuming beverages between meals per day, adequate dietary diversity score and age. |

| [22] | Factors associated with overweight and obesity among adolescents in  Senior High Schools in the  Ga Central  Municipality | Cross-sectional survey | Senior High  School students  (10-19 years) | 219 | 9.59% overweight and  11.42% obese | Female sex, having a slim/slender father or relative, engaging in less or no vigorous physical activity, the number of meals taken in a day. |
| --- | --- | --- | --- | --- | --- | --- |
| [23] | To assess associations between breakfast consumption, BMI-for-Age (BMI), and physical fitness in a cross-section of school-aged children attending government-owned primary schools in Kumasi, Ghana. | Cross-sectional survey | School children | 438 | 10.5% overweight and obese | Female sex |
| [24] | To determine the prevalence and determinants of overweight and obesity among school-age children in two urban settings in Ghana. | Cross-sectional surveys | School children (9-15 years) | 3089 | 17 % overweight and Obese. | Lower participation in sports activity, maternal tertiary education, higher household socioeconomic status, and attending private school. |
| [25] | To determine the predictors  of overweight/obesity among  School-Going Adolescents (SGAs) in Hohoe, Volta region. | Cross-sectional survey | School-Going  Adolescents  (10-19 years) | 400 | 15.1% Overweight/obesity | Daily moderate PA>30 minutes and vigorous PA>60 minutes decreased adolescents’ odds of being overweight/obesity. Playing at school was protective against adolescent overweight/ Obesity. |
| [26] | To assess the dietary and physical activity behaviours and nutritional status of | Cross-sectional survey | School children (8-18 years) | 475 |  | Poor dietary and physical activity |
|  | overweight /obese school children |  |  |  |  |  |
| [27] | To examine the effectiveness of nutrition education on knowledge and BMI-for-age (BFA) of school-aged children in the Kumasi Metropolis | Longitudinal study | School children (9-13 years) | 433 |  | Lack of Physical activity and nutrition knowledge. |
| [28] | To determine associations between dietary patterns and  background characteristics among school-age children (9–15 years) in Ghana. | Cross-sectional survey | School children (9-15 years) | 487 |  | Processed meat, fried foods, and sugar foods. |
| [29] | To examine the association of the schools’ contextual factors with body mass index  (BMI), abdominal obesity and overweight (including obesity) in urban Ghana. | Cross-sectional survey | School children (8-11 years) | 543 | 30.0% overweight | Attending high SES level school, access to healthful foods at school, and after-school recreational facilities. |
